# A case of the first documented fire ant anaphylaxis in Canada

**DOI:** 10.1186/1710-1492-9-25

**Published:** 2013-07-09

**Authors:** Jason Kihyuk Lee, Stephen Daniel Betschel

**Affiliations:** 1 Division of Clinical Immunology and Allergy St Michael’s Hospital, Toronto, Canada

## Abstract

The first documented confirmed case of an imported fire ant causing anaphylaxis in Canada is herein reported. In a patient with anaphylaxis to ants a physician in Canada should be aware that an allergic reaction to fire ant is a possibility.

## Index case

A 43 year old female presented for allergy assessment in December of 2012 having been bitten by several “red ants” in a Toronto park in July of 2012. Her manifestations included immediate localized swelling at the bite sites followed within minutes by generalized pruritus and presyncope. She was taken to a local emergency and treated with intravenous diphenhydramine, ranitidine, and prednisone. It was unclear what the vitals were at the time. Epinephrine was not given and her swelling took a few days to resolve. Her past medical history was significant for mild intermittent asthma and a morphine anaphylaxis. Her physical exam in December 2012 was unremarkable for any findings to suggest urticaria pigmentosa or dermographism.

At the time of the reaction there were no cofactors of anaphylaxis including alcohol, non steroidal anti-inflammatory use, exercise, or proton pump inhibitor use. There were no confounding medications and no prior history of latex or food allergies. Food was not ingested for 4 hours prior to this. This patient is a traveller to Florida and other southern states in the USA but had not been there for at least 4 months.

The standard of care of skin prick testing to whole body prepared fire ants was performed along with the other five venom containing insects in Canada which includes white faced hornet, yellow jacket, yellow hornet, honey bee, and wasp.

To the investigator’s surprise the whole body fire ant commercial extract (ALK 1:100 w/v commercial extract) was positive on skin prick testing with a 6 mm wheal reaction with the positive control being 4 mm wheal and negative skin prick test being 1 mm. The other venoms of the vespids and aphids flying insects were negative at a concentration up to 1 mcg /mL (ALK commercial extracts). Intradermal testing with fire ant was not done as the skin prick test was clearly positive.

## Discussion

Fire ants are endemic to southern and south eastern United States [1] and thus far no previous reports of anaphylaxis to fire ants have been documented in Canada. Once considered simply an outdoor pest limited to a range of a few southern states in the USA, it now appears to be a broader problem with anaphylaxis reported in more northern states [1]. At this time it is unclear the exact mortality rate due to venom from imported fire ants and the extent of this problem in North America.

Several interesting points come out of this fascinating case. It is unclear exactly which factor, be it climate change resulting in warmer winters in Southern Ontario, globalization, or increased trafficking of exotic pets and insects that this patient somehow managed to not only find but have anaphylaxis to imported fire ants. Prior to the winter of 2013 there were no prolonged freezes in Southern Ontario. In fact the presence of fire ants in Canada in multiple provinces and confirmed by Environment Canada has been reported in the Globe and Mail going back to at least 2009 [2]. As physicians the local changes to climate and environment are often overlooked. Most allergists do not routinely have commercially prepared fire ant allergen extracts but if this becomes more of a common occurrence in the future certainly this index case may prompt more physicians to think of this rare cause of anaphylaxis.

It is also possible that there are issues of cross reactivity, however given that all other testing was negative and the absence of scorpion bites in the patient’s history this is unlikely but worthy of considering [3,4].

Moreover, the management of an emergency visit for anaphylaxis should include the administration of adrenaline. In fact this patient personally went out and purchased an epinephrine auto-injector out of pocket highlighting a common problem identified in earlier studies in Canada [5] of inadequate emergency department anaphylaxis action plans. In addition it was noted that the patient initiated the consultation to an allergist by specifically making the request instead of this being part of a routine emergency protocol as is recommended by the American Academy of Allergy Asthma and Immunology (AAAAI) [6] and by Canadian experts on the subject. Admittedly the lack of initiating referrals to allergists is a problem seen across the border with venom anaphylaxis [1,7]. The management would include carrying a MedicAlert® bracelet identifying this allergy and also identifying the fact that this patient carries an EpiPEN® [8]. Furthermore, in anyone with a potential life threatening anaphylaxis to venom with confirmed venom allergy should undergo the standard of care which is desensitization therapy to the venoms [1,6-8].

## Conclusions

Consider reactions to imported fire ants occurring in Southern Ontario as plausible. Routine testing for anaphylaxis in an outdoor setting with an insect may be necessary. We would welcome any other physicians who have noticed this phenomena to contact us.

## Consent

A written informed consent was obtained from the patient for publication of this report using a standard consent form.

## Competing interests

The authors declare that they have no competing interests.

## Authors’ contributions

LJK drafted the manuscript, identified the patient, obtained consent, and responded to all editorial queries. BSD assisted in the review of the manuscript, provided additional references, and assisted in the drafting of the manuscript. Both authors read and approved the final manuscript.
